# Risk factors for birth trauma and postpartum posttraumatic stress in the United Kingdom: Results from the international survey of childbirth‐related trauma

**DOI:** 10.1111/aogs.70236

**Published:** 2026-05-04

**Authors:** Rebecca Webb, Nazihah Uddin, Georgina Constantinou, Susan Ayers

**Affiliations:** ^1^ Centre for Maternal and Child Health Research City, St George's, University of London London UK; ^2^ Institute of Health Sciences Education Queen Mary University of London London UK

**Keywords:** childbirth‐related PTSD, PTSD, risk factors, traumatic birth

## Abstract

**Introduction:**

Recent research shows that 40.6% of women in the United Kingdom experience childbirth as at least moderately traumatic, and 5.9% develop childbirth‐related posttraumatic stress disorder (CB‐PTSD). However, risk factors for birth trauma ratings and CB‐posttraumatic stress symptoms (CB‐PTSS) in this sample remain unexplored. Therefore, the aim was to understand risk factors for birth trauma and CB‐PTSS in a UK sample.

**Material and Methods:**

A cross‐sectional survey of traumatic birth and CB‐PTSD with women (*N* = 339) from three hospitals in England, two in Wales, and three in Scotland. Participants completed the survey at 6–12 weeks postpartum. It included questions about (i) demographics and mental health; (ii) pregnancy; (iii) labor and birth; and (iv) the infant. Outcome variables were birth trauma rating and CB‐PTSS.

**Results:**

Higher birth trauma ratings were predicted by women not having other children, maternal complications during birth, lower birth satisfaction, and a mother not having skin‐to‐skin contact with her baby after birth. Higher CB‐PTSS were predicted by younger maternal age, women not having other children, current mental health difficulties, previous trauma, giving birth in Scotland, emergency cesarean section, and lower birth satisfaction.

**Conclusions:**

Overall, this paper found that traumatic birth and CB‐PTSS symptoms are associated with a range of demographic, mental health, birth‐related, and infant‐related variables. Results from this study can be used to improve maternity care.

## Graphical abstract

Higher birth trauma ratings are predicted by women not having other children, maternal complications, lower birth satisfaction, and no postpartum skin‐to‐skin contact. Higher childbirth‐related post‐traumatic stress symptoms are predicted by younger maternal age, no previous children, current mental health difficulties, previous trauma, emergency cesarean section, and lower birth satisfaction.

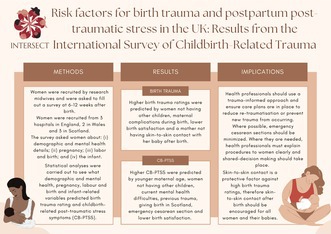

**Abbreviations**
BSS‐R: Birth Satisfaction Scale‐Revised
CB‐PTSD: childbirth‐related post‐traumatic stress disorder
CB‐PTSS: childbirth‐related posttraumatic stress symptoms
City BiTS: City Birth Trauma Scale

**Key message**

Most risk factor studies for CB‐PTSD have been carried out in England. In a sample of women from England, Scotland, and Wales, higher CB‐PTSS are predicted by younger maternal age, not having other children, current mental health difficulties, previous trauma, giving birth in Scotland, emergency cesarean, and lower birth satisfaction.

## 1 INTRODUCTION

Research suggests that approximately 20%–40.6% of women will find childbirth psychologically traumatic.^1^, ^2^, ^3^, ^4^ A traumatic birth experience can have negative effects for women. For example, a meta‐synthesis found that women who had a traumatic birth experienced intense negative emotions, felt a loss of a sense of self and had difficult relationships with their infant and their partner.^5^ Quantitative research has also found women who had a traumatic birth are less likely to initiate breastfeeding or are more likely to breastfeed for a shorter period.^6^ Women are also less likely to have more children in the future^7^ and tend to report lower psychological well‐being.^8^

Reviews and meta‐analyses suggest that 3.1%–4.7% of women will develop childbirth‐related post‐traumatic stress disorder (CB‐PTSD) following a traumatic birth^9^, ^10^, ^11^, ^12^ and recent research carried out in a UK sample found a prevalence of 5.9% for CB‐PTSD.^4^ PTSD is a stress‐related disorder characterized by re‐experiencing symptoms such as flashbacks, avoidance of factors related to the event, negative mood and cognition, and hyperarousal (DSM‐5).^13^

Risk factors for developing CB‐PTSD include pregnancy, labor, and birth and postnatal factors.^14^, ^15^ For example, in a meta‐analysis of 50 studies, pregnancy‐related factors most strongly associated with CB‐PTSD were antenatal depression, fear of childbirth, pregnancy complications, and a history of PTSD. Birth‐related factors most strongly associated with CB‐PTSD were negative subjective birth experiences, having an assisted vaginal birth or a cesarean section, lack of support during labor, and birth and dissociation. After birth, CB‐PTSD was associated with poor coping, stress and depression.^14^

A recent systematic review found risk factors for developing a diagnosis of CB‐PTSD were obstetric interventions and obstetric violence, previous mental illness, previous traumatic experiences, and poor social support throughout pregnancy and/or during birth.^16^ Studies carried out in multiple countries support the findings from the systematic reviews and meta‐analyses. For example, a survey of over 1000 women in Germany found depressive and anxiety symptoms, pregnancy complications, poorer subjective birth experience, low support during birth and unplanned cesarean section predicted CB‐PTSD in women.^17^ Similarly, a study carried out in Norway with 2000 women found high levels of CB‐PTSD were associated with prior PTSD diagnosis, higher fear of childbirth, and worst subjective birth experience.^18^

In studies carried out in England, one cross‐sectional population‐based health survey of 4509 women found that CB‐posttraumatic stress symptoms (CB‐PTSS) were associated with a higher level of deprivation, not having a health professional to talk to about sensitive issues during pregnancy, and the infant being admitted for neonatal intensive care.^19^ In another study carried out with nearly 2000 women, CB‐PTSS symptoms were more common in Black women, women who had a higher pre‐pregnancy BMI, women with a history of mental illness, those who gave birth before arriving at the hospital, those who underwent an emergency cesarean section, or experienced severe maternal morbidity or neonatal complications.^20^

It is clear from the research above that risk factors for developing CB‐PTSD tend to fall within four categories: Women's demographic and mental health history; Pregnancy‐related factors such as complications during pregnancy; Birth‐related factors such as birth method, support during birth; and Infant‐related factors such as infant gestation and neonatal intensive care stay. However, the outcome variables differ across studies (i.e., CB‐PTSD diagnoses vs. CB‐PTSS) and most studies have been limited to England. This research therefore aims to identify which risk factors are associated with birth trauma ratings and CB‐PTSS symptoms in a sample of women recruited from hospitals in England, Scotland, and Wales. This research was part of an international study of CB‐PTSD called INTERSECT (International Survey of Childbirth Related Trauma),^21^ which aims to understand prevalence and risk factors for traumatic birth and CB‐PTSD worldwide.

## 2 MATERIAL AND METHODS

### 2.1 Design

This study was the UK part of the INTERSECT study and was conducted with a cross‐sectional sample of women giving birth in England, Scotland, and Wales.

### 2.2 Participants

Women were recruited through hospitals in England, Wales, and Scotland. Northern Ireland was also part of the research but did not recruit any participants. Three hospitals based in London represent the English sample, three hospitals based in Glasgow represent the Scottish sample, and the Welsh sample is made up of women from a hospital in Powys and a hospital in Swansea. Women were eligible to take part if they were aged 16 or over, had a baby in the previous 6–12 weeks, and were able to provide informed consent. Women were not excluded based on their type of birth or maternal or infant outcomes (including infant death).

### 2.3 Measures

The INTERSECT Questionnaire is made up of nine core measures with a total of 60 questions. This includes questions about pregnancy and birth, birth satisfaction, birth trauma, PTSD, depression and trauma history, and women's demographics.

#### 2.3.1 Dependent variables

##### Birth trauma

Perceived birth trauma was assessed using a single‐item question on a 10‐point scale for women to rate whether they perceived their birth to be traumatic from not at all (0) to extremely (10).

##### CB‐PTSS

The City Birth Trauma Scale^22^ (City BiTS) consists of 29 questions which map onto DSM‐5 diagnostic criteria. Symptoms are rated for frequency over the last week and scored on a scale ranging from 0 (“not at all”) to 3 (“5 or more times”). Scores range from 0 to 60 and a higher score indicates greater symptoms of PTSD. Diagnostic criterion A items are scored on a yes/no scale. Distress, disability, and potential physical causes are rated as yes/no/maybe. The scale can be used as a continuous measure of symptoms (by adding up scores for subscales B–E) or as a diagnostic tool.

#### 2.3.2 Independent variables

##### Demographic and mental health variables

The INTERSECT survey asks questions about basic demographic factors (age, ethnicity, relationship status) and mental health factors such as trauma history, current, and previous mental health diagnoses.

##### Pregnancy‐related variables

The INTERSECT survey asks questions related to the pregnancy, such as whether the pregnancy was a single or multiple pregnancy and about previous pregnancy loss and birth trauma.

##### Birth‐related variables

The INTERSECT survey asks a range of questions related to birth including how women's babies were born, where women gave birth, who was at the birth, type of support offered, and maternal complications during labor and birth.

*Birth satisfaction* was measured using the Birth Satisfaction Scale‐Revised (BSS‐R). The BSS‐R^23^ is a 10‐item, self‐report scale that was reduced from the original 30‐item BSS.^24^ The BSS‐R assesses women's perceptions of birth in order to determine women's satisfaction with their birth experience. The BSS‐R consists of one higher‐order factor (experience of childbirth) containing three lower‐order factors: (1) quality of care provision, measured by four items asking about birth environment, support, and relationships with health professionals; (2) women's personal attributes, measured by two items that ask about women's ability to cope during labor and feeling in control; and (3) stress experienced during labor, measured by four items that ask about distress, long labor, and pain. The BSS‐R is a Likert‐type scale that requests participants to rate their level of agreement with each item (0 = strongly disagree to 4 = strongly agree), with four of its items being reverse‐coded (see Table 1).

**TABLE 1:** Birth Satisfaction Scale‐Revised.^23^

Item	Reverse coded	Subscale
(1) I came through childbirth virtually unscathed	No	Stress experienced during labor
(2) I thought my labor was excessively long	Yes	Stress experienced during labor
(3) The delivery room staff encouraged me to make decisions about how I wanted my birth to progress	No	Quality of care provision
(4) I felt very anxious during my labor and birth	Yes	Women's personal attributes
(5) I felt well supported by staff during my labor and birth	No	Quality of care provision
(6) The staff communicated well with me during labor	No	Quality of care provision
(7) I found giving birth a distressing experience	Yes	Stress experienced during labor
(8) I felt out of control during my birth experience	Yes	Women's personal attributes
(9) I was not distressed at all during labor	No	Stress experienced during labor
(10) The delivery room was clean and hygienic	No	Quality of care provision

*Note*: Participants respond on a 5‐point (0–4) Likert scale based on level of agreement/disagreement (strongly agree; agree; neither agree or disagree; disagree; strongly disagree). Possible scores range from 0 to 40, with higher scores representing higher birth satisfaction.

The INTERSECT UK survey asks additional questions on procedures performed during birth (i.e., episiotomy and Kristeller maneuver). These were taken from the IMAGINE Euro study.^25^

##### Infant variables

The INTERSECT survey asks questions related to infant complications during labor and birth, and the INTERSECT UK survey asks an additional question taken from the IMAGINE Euro survey^25^ regarding whether women were able to have skin‐to‐skin contact with their baby after birth.

### 2.4 Procedure

Research midwives approached eligible women either during antenatal appointments, directly after birth, or during postnatal appointments. Women's details were transferred to the research team who then contacted women 6 weeks after birth with the survey, which could be completed online, via survey host Qualtrics, or using pen and paper. Women were reminded a maximum of three times to complete the survey. Women who completed the survey were entered into a prize draw to win one of four £50 gift vouchers.

### 2.5 Data analysis

Analyses were conducted using the statistics environment SPSS.^26^ The two outcome variables were birth trauma ratings and CB‐PTSS. Birth trauma rating was measured by the 10‐point Likert scale question described above. To assess CB‐PTSS, the sums of subscales B–E of the City BiTS^22^ were totaled. Descriptive statistics were calculated for all variables. Nonparametric tests were carried out to identify independent variables that were significantly associated with the dependent variables using SPSS. To identify significant predictor variables via multiple regression, a data‐driven approach was taken; therefore, the independent variables that were significantly associated with the dependent variables, regardless of level of association, were put into two separate stepwise linear regression models. As belief that women and infants would be seriously injured or die is taken from the City BiTS (i.e. the outcome variable), these were not included in the regression due to concerns over collinearity. CB‐PTSD diagnoses were also calculated and risk factors assessed; however, as only a small number of women met criteria for CB‐PTSD, and therefore due to a lack of statistical power, results for CB‐PTSD are presented in Tables S2–S5.

## 3 RESULTS

### 3.1 Sample

A total of 494 women started the survey, and 366 completed it (68.62%). The final sample included 339 (92.62%) women who completed the survey within the correct time frame. Women were aged 32.12 on average (SD = 5.32), and most women were White (56.0%) and the United Kingdom was their country of birth (72.4%). The majority of women had completed higher education (77.1%), lived in a household with an average income (55.8%) and were married (61.6%). In terms of mental health difficulties, 65.2% had no previous trauma history, and 77.6% of women had no previous mental health difficulties. A small proportion of women (10.0%) had a current psychological or mental health difficulty.

In terms of birth‐related variables, 48.1% of women had a vaginal birth, followed by an emergency cesarean section (23.6%), elective cesarean section (17.4%), and assisted vaginal birth (10.9%). Most women gave birth in hospital in a labor ward or obstetric unit (85.0%), followed by birth center within a hospital (9.1%), at home (3.5%), at a birth center away from a hospital (2.1%), or during transport to their planned place of birth (0.3%). Most women gave birth where they had planned to give birth (82.0%), and care was mostly provided by midwives (68.4%). Some women were given an episiotomy (16.8%) and most women had a partner with them at their birth (97.9%). This was their romantic partner for most women (78.8%).

In terms of infant variables, 82.3% of babies were born without any complications. A total of 8.3% of women thought their baby would be seriously injured during birth, and 7.1% of women thought their baby would die during birth. See Table S1 for more sample characteristics.

### 3.2 Dependent variables

Ninety women (26.5%) perceived their birth to be not at all traumatic. Over 40% of women (40.6%) perceived their birth to be moderately to extremely traumatic. Out of this 40%, 5.0% of women perceived their birth to be extremely traumatic (see Table 2).

**TABLE 2:** Ratings of birth trauma.

Rating	*N* (%)
0 Not at all traumatic	90 (26.5)
1	21 (6.2)
2	36 (10.6)
3	36 (10.6)
4	18 (5.3)
5 Moderately traumatic	51 (15.0)
6	17 (5.0)
7	33 (9.7)
8	14 (4.1)
9	6 (1.8)
10 Extremely traumatic	17 (5.0)

According to DSM‐5 criteria, 20 out of 339 (5.9%) of women met criteria for CB‐PTSD diagnosis. Significant predictors for CB‐PTSD diagnoses were giving birth in Scotland, lower birth satisfaction, emergency cesarean section birth, infant complications, and no skin‐to‐skin contact with the baby following birth (see Table S5).

Total PTSD symptoms ranged from 0 (no symptoms) to 60 (high symptoms) (M = 10.85; SD = 11.59).

### 3.3 Pregnancy‐related variables

According to nonparametric tests, pregnancy‐related variables were not significantly associated with birth trauma ratings or CB‐PTSS.

### 3.4 Demographic and mental health variables

Higher birth trauma ratings were associated with not having had children before (*U* = 10832.00, *p* < 0.001) and living in urban compared to rural areas (KW = 8.535, *p* = 0.014).

Higher CB‐PTSS scores were associated with younger age (*ρ* = −0.127, *p* = 0.020), not having had children before (*U =* 11116.50, *p* < 0.001), having a previous mental health diagnosis (KW = 17.109, *p* < 0.001) or having current mental health problems (KW = 38.953, *p* < 0.001). Women who had experienced previous trauma also had higher CB‐PTSS (*U* = 15883.00, *p* < 0.001), and pairwise comparisons showed women who had experienced multiple traumas had higher CB‐PTSS (*KW* = 33.982, *p* < 0.001) (see Table 3).

**TABLE 3:** Descriptive statistics and frequencies for demographic and mental health variables.

		Birth‐trauma rating	CB‐PTSS
		Mean (SD)	Mean (SD)
Other children	No	**4.23 (3.16)**	**13.60 (13.59)**
	Yes	**2.88 (2.79)**	**8.25 (8.54)**
Previous mental health difficulty	No	3.54 (3.10)	**9.73 (11.20)** ^**a**^
	Don't know	6.00 (3.37)	**19.00 (12.65)**
	Yes	3.38 (2.83)	**14.49 (12.15)** ^**a**^
Current mental health difficulty	No	3.43 (3.02)	**9.10 (10.37)** ^**b**^
	Don't know	4.64 (3.22)	**21.18 (12.58)** ^**b,c**^
	Yes	3.68 (3.06)	**18.71 (13.90)** ^**c**^
Previous trauma	No	3.53 (3.09)	**9.01 (10.59)**
	Yes	3.49 (2.97)	**14.55 (12.80)**
Type of trauma	None	3.56 (3.09)	**9.01 (10.59)** ^**d**^
	Serious life threatening illness	2.14 (1.35)	**4.14 (3.48)** ^**e**^
	Sexual assault	2.42 (2.54)	**12.5 (10.12)**
	Child abuse	2.50 (2.26)	**12.0 (9.42)**
	Accident	4.60 (1.67)	**13.40 (13.69)**
	Natural disaster	4.00 (5.66)	**17.0 (24.04)**
	Other	3.50 (3.12)	**12.91 (12.98)** ^**f**^
	Multiple	4.03 (3.24)	**19.57 (13.30)** ^**d,e,f**^
Living area	City, urban	**3.89 (3.06)** ^**g**^	11.21 (11.52)
	Town, suburban	**3.24 (2.93)**	11.24 (12.59)
	Rural	**2.73 (3.04)** ^**g**^	9.60 (10.52)

*Note*: Bold indicates statistically significant variables; superscript letters indicate significant results for pairwise comparisons.

### 3.5 Birth‐related variables

Higher birth trauma ratings were associated with complications experienced by women during birth (KW = 46.820, *p* < 0.001), and if those complications were still affecting women now (*U* = 3138.00, *p* = 0.006). Women who believed they would be seriously injured (*U* = 11 045.50, *p* < 0.001) or die (*U* = 744.00, *p* < 0.001) during labor and birth had significantly higher birth trauma ratings. Higher birth trauma ratings were also associated with women giving birth by emergency cesarean section or assisted vaginal birth (KW = 48.08, *p* < 0.001), not being able to give birth where planned (*KW* = 15.264, *p* = 0.004), giving birth in a hospital labor ward or obstetric unit (*KW* = 20.195, *p* < 0.001), giving birth in Scotland (*KW* = 12.210, *p* = 0.002) or being taken care of by a professional midwife or nurse midwife compared to an obstetrician or traditional midwife (*KW* = 12.155, *p* = 0.016). Higher birth trauma ratings were also associated with lower birth satisfaction (total BSS score (*ρ* = −0.714, *p* < 0.001), BSS stress (*ρ* = −0.729, *p* < 0.001), BSS personal attributes (*ρ* = −0.638, *p* < 0.001), BSS quality of care (*ρ* = −0.320, *p* < 0.001)).

Higher CB‐PTSS were associated with women experiencing complications during labor and birth (KW = 39.459, *p* < 0.001), and women believing that they would be seriously injured (*U* = 10028.00, *p* < 0.001) or die (*U* = 6775.00, *p* < 0.001) during labor and birth. Higher CB‐PTSS were also associated with women giving birth by emergency cesarean section or assisted vaginal birth (KW = 10.437, *p* = 0.015), being unable to give birth where planned (KW = 16.650, *p* = 0.002), and giving birth in Scotland (*KW* = 9.782, *p* = 0.008). Women who gave birth with multiple birth partners had higher CB‐PTSS compared to women who gave birth with just their romantic partner or a relative (KW = 15.673, *p* = 0.008) (see Table 4). Furthermore, higher CB‐PTSS were associated with lower birth satisfaction (Total: *p* = −0.500, *p* < 0.001; Stress: *p*= −0.492, *p* < 0.001; Quality of care: *ρ* = −0.200, *p* < 0.001; personal attributes: *ρ* = −0.494, *p* < 0.001).

**TABLE 4:** Descriptive statistics and frequencies for birth‐related variables.

		Birth‐trauma rating	CB‐PTSS
		Mean (SD)	Mean (SD)
Maternal complications during pregnancy and birth	No	**2.44 (2.72)** ^**o,p**^	**7.41 (9.13)** ^**b,c**^
	Minor	**4.53 (2.90)** ^**o**^	**14.85 (12.92)** ^**b**^
	Major	**5.03 (3.25)** ^**p**^	**12.03 (11.81)** ^**c**^
Current maternal complications	No	**4.25 (2.85)**	13.82 (12.37)
	Yes	**5.89 (3.08)**	15.40 (13.82)
Perceived threat of injury to self	No	**3.02 (2.79)**	**9.15 (9.86)**
	Yes	**6.84 (2.58)**	**22.20 (15.40)**
Perceived threat of death to self	No	**3.18 (2.83)**	**9.61 (10.11)**
	Yes	**7.43 (2.63)**	**24.61 (17.04)**
Birth method	Vaginal	**3.00 (2.91)** ^**j,k**^	**8.66 (9.29)** ^**a,b**^
	Assisted vaginal	**5.14 (2.86)** ^**l**^	**13.00 (10.56)** ^**b**^
	Emergency cesarean	**4.98 (2.30)** ^**j,m**^	**14.70 (16.28)** ^**a**^
	Elective cesarean	**2.03 (2.33)** ^**k,l,m**^	**10.39 (8.70)**
Planned place	Yes	**3.23 (2.95)** ^**q**^	**9.89 (10.90)** ^**d**^
	No, couldn't reach planned place of care	**5.67 (5.13)**	**5.67 (4.04)**
	No, moved from birth center to labor ward	**5.14 (3.18)** ^**q**^	**21.46 (15.91)** ^**d,e**^
	No, transferred from home/birth center to hospital on medical advice	**4.53 (3.06)**	**10.18 (7.82)** ^**e**^
	No, transferred from home/birth center for other reason	**4.69 (2.98)**	**10.54 (9.40)**
Actual place	Hospital labor ward or obstetric unit	**3.82 (3.06)** ^**r,s**^	11.48 (12.10)
	Birth center or midwifery unit	**2.32 (2.59)** ^**s**^	8.50 (8.04)
	Free standing community clinic/birth center	**1.71 (1.98)**	6.71 (6.02)
	At home	**1.17 (2.17)** ^**r**^	5.00 (4.33)
	During transport	**0.00**	0.00
Devolved nation	England	**3.95 (3.04)** ^**t**^	**10.68 (11.67)** ^**f**^
	Scotland	**3.53 (3.11)**	**16.71 (14.10)** ^**f,g**^
	Wales	**2.69 (2.88)** ^**t**^	**8.98 (9.61)** ^**g**^
Main health professional carer during birth	Obstetrician or physician	**2.83 (2.73)** ^**u**^	11.28 (11.61)
	Professional midwife or nurse midwife	**3.88 (3.11)** ^**u,v**^	10.73 (12.15)
	Community health worker or nurse	**1.00 (1.41)**	2.50 (3.53)
	Traditional midwife	**2.69 (2.90)** ^**v**^	11.29 (8.90)
	Other	**5.50 (0.71)**	12.00 (8.48)
Birth partner	Partner	3.51 (3.08)	**10.06 (11.17)** ^**h**^
	Friend	0.00	**3.00 (no SD)**
	Relative	3.33 (3.14)	**8.25 (12.13)** ^**i**^
	Other	0.00	**12.00 (no SD)**
	No other	2.71 (3.45)	**7.86 (8.97)**
	Multiple	3.96 (2.82)	**16.12 (12.89)** ^**h,i**^

*Note*: Bold indicates statistically significant variables, superscript letters indicate significant results for pairwise comparisons.

### 3.6 Infant‐related variables

Higher birth trauma ratings and higher CB‐PTSS were associated with infant complications, and women perceiving that their infant would be seriously injured or would die during labor and birth. Skin‐to‐skin contact immediately after birth appeared to be a protective factor and was associated with lower birth trauma ratings and CB‐PTSS (Table 5).

**TABLE 5:** Descriptive statistics and frequencies for infant‐related variables.

		Birth‐trauma rating		CB‐PTSS	
		Mean (SD)		Mean (SD)	
Infant complications	No	3.25 (3.00)^d^	*KW* = 14.624, *p* < 0.001	9.84 (11.21)^a,b^	*KW* = 16.521, *p* < 0.001
	Yes, minor	4.94 (2.98)^d^		15.11 (12.25)^a^	
	Yes, major	4.00 (2.37)		19.33 (12.61)^b^	
Maternal fear her baby would be seriously injured	No	3.20 (2.85)	*U* = 7319.0, *p* < 0.001	9.37 (9.82)	*U* = 7224.50, *p* < 0.001
	Yes	9.37 (9.82)		27.29 (16.28)	
Maternal fear her baby would die	No	3.26 (2.91)	*U* = 6207.00, *p* < 0.001	9.65 (10.18)	*U* = 6109.00, *p* < 0.001, ratings
	Yes	7.04 (2.63)		26.54 (16.73)	
Skin to skin	Yes	3.24 (2.91)^e,f^	*KW* = 20.940, *p* < 0.001	10.04 (10.58)^c^	*KW* = 11.629, *p* = 0.003
	No	6.15 (3.78)^e^		26.54 (20.47)^c^	
	No opportunity due to medical reasons	5.72 (2.81)^f^		14.48 (11.60)	

*Note*: All results are significant; superscript letters indicate significant results in pairwise comparisons.

### 3.7 Predictor variables

#### 3.7.1 Birth trauma rating

Significant variables from the bivariate analyses put into the regression model for birth trauma were whether women had other children, birth satisfaction score, maternal complications, and skin‐to‐skin contact with their infant after birth.

The model had a Durbin Watson Score of 1.88, thus suggesting the assumption of independent error was met. This model was significant (*F* = 96.44, *p* < 0.001) and explained 54.3% of the variation (*R* ^2^ = 0.543). Significant predictors for a higher birth trauma rating were lower birth satisfaction (*p* < 0.001), not having other children (*p* = 0.022), maternal complications (*p* = 0.002), and not having skin‐to‐skin contact with the baby after birth (*p* = 0.004).

#### 3.7.2 CB‐PTSS

Significant variables from the bivariate analyses that were put into the regression model for CB‐PTSS were maternal age, whether women had other children, current mental health difficulties, previous trauma, devolved nation, birth satisfaction score, and birth method. The model had a Durbin Watson Score of 1.76, thus suggesting the assumption of independent error was met. This model was significant (*F* = 32.383, *p* < 0.001) and explained 43.2% of the variation (*R* ^2^ = 0.432).

Significant predictors for higher CB‐PTSS were younger maternal age (*p* = 0.005), women not having other children (*p* = 0.006), current mental health difficulties (*p* ≤ 0.001), previous trauma (*p* = 0.008), giving birth by emergency cesarean section (*p* = 0.049), giving birth in Scotland (*p* = 0.024), and lower birth satisfaction (*p* < 0.001).

The plot of the residuals showed they were not normally distributed therefore the square root for the total CB‐PTSS variable was calculated and the regression model rerun. The model was also significant (*x* ^2^ = 31.50, *p* < 0.001), the predictor variables remained the same, and the variance explained was similar (41.1%) (Table 6).

**TABLE 6:** Regression output for three outcome variables (CB‐PTSS; birth trauma rating) showing variables that significantly contributed to the final model.

	Birth trauma rating	CB‐PTSS
	B: 95 CI (*p*)	B: 95 CI (*p*)
Maternal age	–	−0.277: −0.472 to −0.08 (0.005)
Other children	−0.536: −0.994 to −0.078 (0.022)	−2.829: −4.86 to −0.80 (0.006)
Current mental health difficulty	–	3.247: 1.53–4.96 (<0.001)
Previous trauma	–	3.069: 0.814–5.32 (0.008)
Devolved nation	–	1.767: 0.239–3.29 (0.024)
BSS total	−0.255: −0.287 to −0.233 (<0.001)	−0.785: −0.918 to −0.651 (<0.001)
Birth method	–	0.852: 0.003–1.70 (0.049)
Maternal complications	0.576: 0.211–0.940 (0.002)	–
Infant complications	–	–
Skin‐to‐skin contact	0.608: 0.190–1.03 (0.004)	–

## 4 DISCUSSION

The aim of this study was to identify key risk factors associated with birth trauma ratings and CB‐PTSS in a UK sample. The results show that there were many bivariate associations that are broadly consistent with previous research.^14^, ^15^, ^16^, ^17^, ^18^, ^19^, ^20^ The results showed that pregnancy‐related variables, such as whether the mother gave birth to one or multiple babies, previous pregnancy loss, and previous birth trauma were not associated with birth trauma ratings or CB‐PTSS. This is in line with previous research which has found fear of childbirth, complications during pregnancy, and poor social support during pregnancy are associated with CB‐PTSD.^14^, ^16^, ^17^

The multivariate models highlight the key predictors of the outcomes. In terms of birth trauma ratings, not having other children, maternal complications, lower birth satisfaction, and women not having skin‐to‐skin contact with their baby immediately after birth were associated with higher birth trauma ratings. In terms of CB‐PTSS, younger maternal age, not having other children, having a current mental health difficulty, experiencing previous trauma, giving birth in Scotland, giving birth by emergency cesarean section, and lower birth satisfaction were associated with higher CB‐PTSS. Predictors of CB‐PTSD were similar, with giving birth in Scotland, lower birth satisfaction, emergency cesarean section birth, infant complications, and no skin‐to‐skin contact with the baby following birth identified as significant predictors. However, due to the low sample size of women with CB‐PTSD, these findings may be less reliable.

The findings from the study are in line with previous research that has found age to be a significant predictor for CB‐PTS (both CB‐PTSD and CB‐PTSS),^27^, ^28^ as is having mental health difficulties and having experienced previous traumatic events.^15^ The finding that being a first‐time mother was associated with CB‐PTSS has also been found in a meta‐analysis of CB‐PTS^14^ and could be related to research that has found feeling empowered and having a sense of trust can reduce whether women find birth traumatic.^29^ Therefore, having previous experiences of giving birth may contribute to this feeling of trust in oneself.

In terms of birth‐related variables, higher birth satisfaction was a significant predictor of lower birth trauma rating and lower CB‐PTSS. In this study, birth satisfaction was measured using the Birth Satisfaction Scale^24^ and is made up of three subscales relating to the quality‐of‐care provision; stress during labor and women's personal attributes. Factors related to these subscales have been found to be associated with CB‐PTS before such as being communicated well with by staff, women being able to make their own decisions,^30^, ^31^, ^32^ having a distressing birth experience,^14^ and feeling out of control during birth,^15^, ^33^, ^34^ therefore it makes sense that CB‐PTS is associated with birth satisfaction. Furthermore, previous research has found an association between birth satisfaction and CB‐PTS.^35^, ^36^

Giving birth by emergency cesarean section was associated with higher birth trauma ratings and CB‐PTSS. This finding is in line with previous research which has found that emergency cesarean sections increase the risk of developing CB‐PTSD.^14^, ^16^, ^17^, ^20^, ^37^ Qualitative research suggests that women who have emergency cesarean sections experience fear, shock, and de‐realization.^38^, ^39^ This therefore suggests the importance of health professionals explaining procedures to women clearly and providing reassurance and support.

The devolved nation women gave birth in was also a predictor of CB‐PTSS, with women who gave birth in Scotland having higher CB‐PTSS than those who gave birth in England and Wales. The reasons for this are unknown and may be due to differences in sample characteristics, obstetric intervention rates, or maternity care. Therefore, future research should look at CB‐PTSS in a wider sample of women in Scotland and other devolved nations to try and ascertain the reasons behind this finding.

In terms of infant‐related variables, the findings showed that not having skin‐to‐skin contact between a mother and her baby after birth predicted birth trauma ratings. Research has found that skin‐to‐skin contact encourages oxytocin release,^40^ which can counteract biological stress responses.^41^ It could therefore be suggested that skin‐to‐skin contact following birth may act as a protective mechanism against how traumatic a woman found her birth to be; however, more research is needed.

The findings suggest that demographic‐, birth‐, and infant‐related factors predict birth trauma ratings and CB‐PTSS. These findings can be used to prevent or treat birth trauma and CB‐PTSS. For example, results suggest that women who are more vulnerable, for example, younger and with current mental health difficulties, are more at risk of higher CB‐PTSS. The use of a trauma‐informed approach to care has been suggested as a way to improve care for women.^42^ One of the key principles is for health professionals to be aware of the widespread nature of trauma and that pregnancy and birth can be a time during which previous trauma can be re‐triggered. Health professionals should therefore ensure care plans are in place to reduce re‐traumatization or prevent new trauma from occurring.^42^

Another of the findings was that emergency cesarean births can increase CB‐PTSS. While these operations can be lifesaving, it is important that emergency cesarean sections are minimized as much as possible. Where they are needed, health professionals must explain procedures to women clearly. Research suggests that CB‐PTSD can be associated with health professionals performing procedures without consent and not communicating well with women,^30^, ^31^, ^32^ therefore clear communication and shared decision‐making between women and health professionals may be a way to prevent CB‐PTSD from developing. While it should be acknowledged that there are time constraints in emergency situations which may prevent shared decision‐making, conversations with health professionals about women's choices and birth options should start during pregnancy^43^ to prepare women for all eventualities.

Another finding identified by this study is that skin‐to‐skin contact is a protective factor against high birth trauma ratings. It is therefore important that skin‐to‐skin contact after birth is encouraged for all women and their babies.

This paper has provided information on risk factors related to birth trauma ratings and CB‐PTSS in a community‐based sample of women. The strengths of this paper include that it is part of the INTERSECT study where multiple different countries have used the same validated measures, meaning the results from the Unite Kingdom can be compared on an international level. It has used a rigorous recruitment method, reducing the bias of recruiting online and recruiting a diverse sample of women, with 56.0% of the sample being White. However, there are some limitations of this research; for example, there was a low completion rate (68.62%) which could suggest bias. Possibly related to this is that only a small proportion of women in our sample (5.9%) had CB‐PTSD, thus meaning there was less statistical power when comparing between groups of women with and without CB‐PTSD diagnoses and is why these data are only presented in Supporting Information. Research with a larger sample is needed to identify risk factors for diagnoses of CB‐PTSD.

## 5 CONCLUSION

Overall, this paper found that birth trauma ratings and CB‐PTSS are associated with a range of demographic, mental health, birth‐related, and infant‐related variables. Results from this study can be used to improve maternity care through trauma informed care, explanations of interventions to women, and shared decision‐making, and promotion of skin‐to‐skin contact between women and their babies.

## AUTHOR CONTRIBUTIONS

RW contributed to protocol development, oversaw ethical approval, supervised NU & GC, helped with recruitment and data collection, carried out data analysis, and prepared the manuscript. NU helped with recruitment and data collection, provided feedback on the manuscript. GC helped with protocol development, provided feedback on the manuscript. SA conceptualized the study and oversaw the project.

## CONFLICT OF INTEREST STATEMENT

There are no conflicts of interest to report.

## ETHICS STATEMENT

Ethical approval was received from West of Scotland Research Ethics Committee on September 1, 2023 (22/WS/0066). All women provided informed consent before completing the survey.

## Supporting information


**Table S1.** Demographic, mental health, and birth and infant variables.
**Table S2**. Demographic and mental health variables.
**Table S3**. Birth‐related variables.
**Table S4**. Infant‐related variables.
**Table S5**. Predictor variables for CB‐PTSD diagnoses.

## Data Availability

The data that support the findings of this study are available on request from the corresponding author. The data are not publicly available due to privacy or ethical restrictions.
