# Supplementary material for: Risk factors for birth trauma and postpartum posttraumatic stress in the United Kingdom: Results from the international survey of childbirth‐related trauma
Source: Acta Obstet Gynecol Scand. 2026 May 4;105(6):1163–72. doi: 10.1111/aogs.70236 (PMC13191812; doi:10.1111/aogs.70236)
Supplement: Supplementary file 1 — Table S1. Demographic, mental health, and birth and infant variables. Table S2. Demographic and mental health variables. Table S3. Birth‐related variables. Table S4. Infant‐related variables. Table S5. Predictor variables for CB‐PTSD diagnoses. [file AOGS-105-1163-s001.docx]

**Risk factors for birth trauma, CB-PTSS and CB-PTSD – Supplementary materials**

**Table S1.** Demographic, mental health, birth and infant variables

|  | **M (SD)** | **Range** |
| --- | --- | --- |
| **Age** | 32.13 (5.32) | 18-48 |
|  | **Answer option** | **N (%)** |
| **Ethnicity** | Ethnic/racial majority (White) | 188 (56.0) |
|  | Ethnic/racial minority | 65 (19.3) |
|  | Not sure | 83 (24.5) |
| **Living in country of birth** | No | 93 (27.6) |
|  | Yes | 244 (72.4) |
| **Living area** | City, urban | 187 (55.7) |
|  | Town, sub-urban | 87 (25.9) |
|  | Rural area | 62 (18.5) |
| **Education** | No formal education | 8 (2.4) |
|  | Primary school | 5 (1.5) |
|  | Secondary school | 64 (19.0) |
|  | Higher education | 259 (77.1) |
| **Income** | Below average | 34 (10.1) |
|  | Average | 187 (55.8) |
|  | Above average | 114 (34.0) |
| **Relationship status** | Married or civil partnership | 207 (61.6) |
|  | Living with partner | 88 (26.2) |
|  | In a relationship but not living together | 16 (4.7) |
|  | Single/Separated or divorced | 25 (7.4) |
| **Do you have any other children (not including this one?)** | Yes | 175 (51.6) |
|  | No | 164 (48.4) |
| **Other children** | 1 | 119 (35.1) |
|  | 2 | 44 (13.0) |
|  | 3 | 6 (1.8) |
| **Previous trauma history** | No trauma | 221 (65.2) |
|  | Serious or life-threatening illness | 7 (2.1) |
|  | Sexual assault | 12 (3.5) |
|  | Child abuse | 6 (1.8) |
|  | Accident | 5 (1.5) |
|  | Other (including physical assault and natural disaster to preserve anonymity) | 47 (13.9) |
|  | Multiple | 35 (10.3) |
| **Previous diagnosis of mental health difficulty** | Yes | 72 (21.2) |
|  | Don’t know | 4 (1.2) |
|  | No | 263 (77.6) |
| **Current psychological or mental health problem** | Yes | 34 (10.0) |
|  | Don’t know | 22 (6.5) |
|  | No | 283 (83.5) |
| **Have you received treatment for your mental health problem?** | Yes currently | 27 (8.0) |
|  | Yes in the past | 46 (13.6) |
|  | No | 7 (2.1) |
| **Current help for mental health problem** | Medication | 7 (2.1) |
|  | Support from professional (e.g. talking therapy) | 11 (3.2) |
|  | Both | 9 (2.7) |
|  | **Answer option** | **N (%)** |
| **Number of babies born in recent birth** | 1 baby | 334 (98.5) |
|  | Multiple babies (including twins and more than 2 babies to preserve anonymity) | 5 (1.5) |
| **Would you consider the birth of your other children traumatic** | No | 246 (72.6) |
|  | Yes | 84 (24.8) |
| **Have you experienced pregnancy loss?** | No | 225 (66.4) |
|  | Yes | 112 (33.0) |
|  | **Answer option** | **N (%)** |
| **How was your baby born?** | Vaginal birth | 163 (48.1) |
|  | Assisted vaginal birth | 37 (10.9) |
|  | Emergency cesarean section | 80 (23.6) |
|  | Elective cesarean | 59 (17.4) |
| **Did you have any medical complications during pregnancy/birth** | No | 170 (50.1) |
|  | Yes, minor complications | 137 (40.4) |
|  | Yes, major complications | 32 (9.4) |
| **Are these complications still affecting you?** | No | 138 (40.7) |
|  | Yes | 35 (10.3) |
| **Where did you have your baby?** | In hospital in a labor ward or obstetric unit | 288 (85.0) |
|  | In a birth center or midwifery-led unit within a hospital | 31 (9.1) |
|  | In a community clinic or birth center away from the hospital | 7 (2.1) |
|  | At home or during transport to preserve anonymity | 13 (3.8) |
| **Was that where you planned to have your baby?** | Yes | 278 (82.0) |
|  | No – I had my baby at home because I could not reach my planned place of care | 3 (0.9) |
|  | No – I moved from the hospital birth center/midwifery unit to the hospital labor ward | 28 (8.3) |
|  | No – I transferred to hospital from home or birth center due to medical advice | 17 (5.0) |
|  | No – I transferred to hospital from home or birth center for another reason (e.g. pain relief) | 13 (3.8) |
| **Who provided most of your care?** | Obstetrician or physician | 54 (15.9) |
|  | Professional midwife or nurse midwife | 232 (68.4) |
|  | Community health worker or nurse | 2 (0.6) |
|  | Traditional midwife | 49 (14.5) |
|  | Other | 2 (0.6) |
| **Continuity of care** | Usually saw the same person or few people | 136 (40.1) |
|  | Usually saw people from the same team | 83 (24.5) |
|  | Usually saw different people at each visit | 120 (35.4) |
| **Was an episiotomy performed?** | Yes | 57 (16.8) |
|  | No | 257 (75.8) |
|  | Don’t know | 24 (7.4) |
| **Kristeller manoeuvre** | Yes | 20 (5.9) |
|  | No | 283 (83.5) |
|  | Don’t know | 36 (10.6) |
| **During your birth did you suffer from any forms of abuse?** | Yes, always/nearly always | 7 (2.1) |
|  | Sometimes | 27 (8.0) |
|  | No, never/almost never | 305 (90.0) |
| **Type of abuse** | Physical abuse | 3 |
|  | Verbal abuse | 4 |
|  | Emotional abuse | 14 |
|  | Other type of abuse | 5 |
|  | Multiple types of abuse | 8 |
| **During labor and birth, did you believe you would be seriously injured?** | No | 294 (86.7) |
|  | Yes | 45 (13.3) |
| **During labor and birth, did you believe you would die?** | No | 311 (91.7) |
|  | Yes | 28 (8.3) |
| **Who was with you at your birth?** | Partner | 267 (78.8) |
|  | Relative | 12 (3.5) |
|  | Other, including friend to preserve anonymity | 2 (0.6) |
|  | No one | 7 (2.1) |
|  | More than one person | 51 (15.0) |
| **How much support did your birth partner give you?** | None – a little, to preserve anonymity | 19 (5.6) |
|  | A moderate amount | 31 |
|  | A lot | 79 |
|  | A great deal | 203 |
| **Devolved nation** | England | 201 (59.3) |
|  | Wales | 100 (29.5) |
|  | Scotland | 38 (11.2) |
|  | **M (SD)** | **Range** |
| **Birth satisfaction scale - Stress** | 8.55 (4.05) | 0-16 |
| **Birth satisfaction scale – Personal attributes** | 3.94 (2.23) | 0-8 |
| **Birth satisfaction scale – Quality of care** | 12.62 (2.94) | 2-16 |
| **Birth satisfaction scale - Total** | 25.07 (7.59) | 4-40 |
|  | **M (SD)** | **Range** |
| **Gestation when born** | 38.95 (1.63) | 33-43 |
|  | **Answer option** | **N (%)** |
| **Did your baby have any medical complications?** | No | 279 (82.3) |
|  | Yes, minor | 54 (15.9) |
|  | Yes, major | 6 (1.8) |
| **Are these complications still affecting your baby?** | No | 54 |
|  | Yes | 10 |
| **In the first hour after birth, did you have skin-to-skin** | Yes | 301 (88.8) |
|  | No | 13 (3.8) |
|  | No opportunity to do so (e.g. baby admitted to NICU) | 25 (7.4) |
| **During labor and birth did you believe your baby would be seriously injured?** | No | 311 (91.7) |
|  | Yes | 28 (8.3) |
| **During labor and birth did you believe your baby would die?** | No | 315 (92.9) |
|  | Yes | 24 (7.1) |

**Results for CB-PTSD Diagnoses**

**Demographic and mental health variables**

Younger age (*U =* 2297.50, *p* = .039), not having other children (*X^2^*(1) = 9.979, *p* = .046), having a previous mental health diagnosis (*X^2^*(2) = 9.49, *p* = .009) were all associated with CB-PTSD diagnosis (see Table 2).

**Table S2**. Demographic and mental health variables

|  |  | No CB-PTSD Diagnosis  N (%) | Yes CB-PTSD Diagnosis  N (%) |
| --- | --- | --- | --- |
| Age |  | **M = 32.39; SD = 5.25** | **M = 29.5; SD = 5.77** |
| Other children | No | **150 (47.0)** | **14 (70.0)** |
|  | Yes | **169 (53.0)** | **6 (30.0)** |
| Previous mental health difficulty | No | **247 (77.4)** | **16 (80.0)** |
|  | Don’t know | **4 (1.3)** | **0 (0.0)** |
|  | Yes | **68 (21.3)** | **4 (20.0)** |
| Current mental health difficulty | No | 271 (85.0) | 12 (60.0) |
|  | Don’t know | 18 (5.6) | 4 (20.0) |
|  | Yes | 30 (9.4) | 4 (20.0) |
| Previous trauma | No | 209 (66.8) | 12 (60.0) |
|  | Yes | 104 (33.2) | 8 (40.0) |
| Type of trauma | None | 209 (66.8) | 12 (60.0) |
|  | Serious life threatening illness | 7 (2.2) | 0 (0.0) |
|  | Sexual Assault | 12 (3.8) | 0 (0.0) |
|  | Child abuse | 6 (1.9) | 0 (0.0) |
|  | Accident | 5 (1.6) | 0 (0.0) |
|  | Natural disaster | 2 (0.6) | 0 (0.0) |
|  | Other | 40 (12.8) | 4 (20.0) |
|  | Multiple | 31 (9.9) | 4 (2.00) |
| Living area | City, urban | 177 (56.0) | 10 (50.0) |
|  | Town, sub-urban | 81 (25.6) | 6 (30.0) |
|  | Rural | 58 (18.4) | 4 (20.0) |

**Birth variables**

Giving birth by emergency cesarean section (*X^2^*(3) = 16.184, *p* = .001), maternal complications during birth (*X^2^*(2) = 7.73, *p* = .021), perceived threat of injury to self (*X^2^*(1) = 59.407, *p* <.001), perceived threat of death to self (*X^2^*(1) = 37.86, *p* <.001), not being able to give birth where planned (*X^2^* (4) = 10.017, *p* = .040), giving birth in Scotland (*X^2^*(2) = 7.57, *p* = .023) and lower birth satisfaction (*U* = 976.50, *p* <.001) were all associated with CB-PTSD diagnoses (see Table 3).

**Table S3**. Birth related variables

|  |  | No CB-PTSD Diagnosis  M (SD) | Yes CB-PTSD Diagnosis  M (SD) |
| --- | --- | --- | --- |
| BSS Stress subscale | | **8.86 (3.90)** | **3.60 (2.87)** |
| BSS Personal Attributes subscale | | **4.10 (2.17)** | **1.40 (1.54)** |
| BSS Quality of care subscale | | **12.72 (2.89)** | **11.05 (3.27)** |
| BSS Total score | | **25.65 (7.32)** | **16.05 (5.98)** |
|  | | No CB-PTSD Diagnosis  N (%) | Yes CB-PTSD Diagnosis  N (%) |
|  | |  |  |
| Birth method | Vaginal | **158 (49.5)** | **5 (25.0)** |
|  | Assisted vaginal | **35 (11.0)** | **2 (10.0)** |
|  | Emergency cesarean | **68 (21.3)** | **12 (60.0)** |
|  | Elective cesarean | **58 (18.2)** | **1 (5.0)** |
| Maternal complications during pregnancy and birth | No | **166 (52.0)** | **4 (20.0)** |
|  | Minor | **124 (38.9)** | **12 (65.0)** |
|  | Major | **29 (9.1)** | **3 (15.0)** |
| Current maternal complications | No | 125 (79.6) | 13 (81.3) |
|  | Yes | 32 (20.4) | 3 (18.8) |
| Perceived threat of injury to self | No | **288 (90.3)** | **6 (30.0)** |
|  | Yes | **31 (9.7)** | **14 (70.0)** |
| Perceived threat of death to self | No | **300 (94.0)** | **11 (55.0)** |
|  | Yes | **19 (6.0)** | **9 (45.0)** |
| Planned place | Yes | **265 (83.1)** | **13 (65.0)** |
|  | No, couldn’t reach planned place of care | **3 (0.9)** | **0 (0)** |
|  | No, moved from birth center to labor ward | **23 (7.2)** | **5 (25.0)** |
|  | No, transferred from home/birth center to hospital on medical advice | **15 (4.7)** | **2 (10.0)** |
|  | No, transferred from home/birth center for other reason | **13 (4.1)** | **0 (0)** |
| Actual place | Hospital labor ward or obstetric unit | 269 (84.3) | 19 (95.0) |
|  | Birth center or midwifery unit | 30 (9.4) | 1 (5.0) |
|  | Free standing community clinic/birth center | 7 (2.2) | 0 (0) |
|  | At home | 12 (3.8) | 0 (0) |
|  | During transport | 1 (0.3) | 0 (0) |
| Devolved nation | England | **192 (60.2)** | **9 (45.0)** |
|  | Scotland | **32 (10.0)** | **6 (30.0)** |
|  | Wales | **95 (29.8)** | **5 (25.0)** |
| Main health professional carer during birth | Obstetrician or physician | 52 (16.3) | 2 (10.0) |
|  | Professional midwife or nurse midwife | 214 (67.1) | 18 (90.0) |
|  | Community health worker or nurse | 2 (0.6) | 0 (0) |
|  | Traditional midwife | 49 (15.4) | 0 (0) |
|  | Other | 2 (0.6) | 0 (0) |
| Birth partner | Partner | 252 (79.3) | 14 (70.0) |
|  | Friend | 1 (0.3) | 0 (0) |
|  | Relative | 12 (3.8) | 0 (0) |
|  | Other | 1 (0.3) | 0 (0) |
|  | No other | 7 (2.2) | 0 (0) |
|  | Multiple | 45 (14.1) | 6 (30.0) |

**Infant-related variables**

Infant complications (*X^2^*(2) = 14.91, *p* <.001), perceived infant injury (*X^2^*(1) = 48.81, *p* <.001), perceived infant death: (*X^2^*(1) 25.185, *p* <.001), and not having skin-to-skin contact (*X^2^*(2) = 26.38, *p* <.001) with the baby after birth were all associated with CB-PTSD diagnoses (see Table 4)

**Table S4.** Infant related variables

|  |  | No CB-PTSD Diagnosis  N (%) | Yes CB-PTSD Diagnosis  N (%) |
| --- | --- | --- | --- |
| Infant complications | No | 268 (84.0) | 11 (55.0) |
|  | Yes, minor | 47 (14.7) | 7 (35.0) |
|  | Yes, major | 4 (1.3) | 2 (10.0) |
| Maternal fear her baby would be seriously injured | No | 301 (94.4) | 10 (50.0) |
|  | Yes | 18 (5.6) | 10 (50.0) |
| Maternal fear her baby would die | No | 302 (94.7) | 13 (65.0) |
|  | Yes | 17 (5.3) | 7 (35.0) |
| Skin to skin | Yes | 288 (90.3) | 13 (65.0) |
|  | No | 8 (2.5) | 5 (25.0) |
|  | No opportunity due to medical reasons | 23 (7.2) | 2 (10.0) |

**CB-PTSD Diagnoses predictor variables**

Significant variables from the bivariate analyses put into the regression model for CB-PTSD were: devolved nation, birth satisfaction score, birth method, infant complications and skin-to-skin contact after birth. The overall model was statistically significant, (χ2 (10) = 68.259, p <.001) and explained between 18.6 (Cox & Snell) to 50.8% (Nagelkerke) of the variation. Significant predictors were birth-satisfaction, birth method, devolved nation, infant complications and skin-to-skin contact. The odds ratio for birth-satisfaction was .818, showing that the probability of having CB-PTSD diagnoses decreases with higher birth-satisfaction. The way women gave birth was also a significant predictor (p = .026). The odds ratios show the probability of having CB-PTSD is 3.88 times higher in an emergency cesarean section birth compared to a vaginal birth (p = .061). The devolved nation women gave birth in was also a significant predictor (p = .001). The odds ratios showed that the odds of developing CB-PTSD are 18.28 times higher in Scotland, compared to England (p <.001). The infant having complications during pregnancy or birth was also a significant predictor (p = .002). The odds of developing CB-PTSD diagnoses were 163.10 times higher in infants who had major complications, compared to infants with no complications (p <.001). Lastly, skin-to-skin contact after birth was a significant predictor (p = .018). The odds of developing CB-PTSD were 8.55 times higher in women who did not have skin-to-skin contact with their baby after birth compared to those who did (p = .018) (see Table 5).

**Table S5.** Predictor variables for CB-PTSD Diagnoses

|  | **CB-PTSD Diagnoses**  **Wald (p)** |
| --- | --- |
| Maternal age | - |
| Other children | - |
| Current mental health difficulty | - |
| Previous trauma | - |
| Devolved nation | 13.094 (.001) |
| BSS Total | 16.00 (<.001) |
| Birth method | 9.254 (.026) |
| Maternal complications | - |
| Infant complications | 12.696 (.002) |
| Skin-to-skin contact | 8.060 (.018) |
